# Effects of physical activity on colorectal cancer risk among family history and body mass index subgroups: a systematic review and meta-analysis

**DOI:** 10.1186/s12885-017-3970-5

**Published:** 2018-01-11

**Authors:** Eileen Shaw, Megan S. Farris, Chelsea R. Stone, Jeroen W. G. Derksen, Rhys Johnson, Robert J. Hilsden, Christine M. Friedenreich, Darren R. Brenner

**Affiliations:** 1 Department of Cancer Epidemiology and Prevention Research, Cancer Control Alberta, Alberta Health Services, Holy Cross Centre, Room 513C, Box ACB, 2210 2nd Street S.W., Calgary, AB T2S 3C3 Canada; 2 Department of Community Health Sciences, Cumming School of Medicine, University of Calgary, Calgary, AB Canada; 3 Department of Oncology, Cumming School of Medicine, University of Calgary, Calgary, AB Canada; 4 Forzani & MacPhail Colon Cancer Screening Centre, Alberta Health Services, Calgary, AB Canada

**Keywords:** Exercise, Colorectal neoplasms, Body mass index, Family history, Risk

## Abstract

**Background:**

Physical activity is consistently associated with a reduced risk of colorectal cancer in epidemiologic studies. This association among higher risk subgroups, such as those with a first-degree family history of colorectal cancer or high body mass index remains unclear.

**Methods:**

We searched MEDLINE for studies examining physical activity and colorectal cancer risk among higher risk subgroups through July 11, 2017. Fifteen and three studies were eligible for inclusion for body mass index and first-degree family history of colorectal cancer subgroups, respectively. Estimates of the highest to lowest comparison of physical activity for each subgroup of risk were pooled using random-effects models.

**Results:**

The pooled associations of physical activity and colorectal cancer risk for those without and with a first-degree family history of colorectal cancer were 0.56 (95% confidence interval (CI) = 0.39–0.80) and 0.72 (95% CI = 0.39–1.32), respectively (p_heterogeneity_ = 0.586). The pooled associations of physical activity and colorectal cancer risk for the low and high body mass index groups were 0.74 (95% CI = 0.66–0.83) and 0.65 (95% CI = 0.53–0.79), respectively (p_heterogeneity_ = 0.389).

**Conclusions:**

Overall, a stronger relative risk of physical activity on colorectal cancer risk was observed in the higher body mass index group, although the difference was not statistically significant, suggesting an added benefit of physical activity as a cancer prevention strategy in population groups with strong risk factors for colorectal cancer. Additional research among these subgroups is warranted.

**Electronic supplementary material:**

The online version of this article (10.1186/s12885-017-3970-5) contains supplementary material, which is available to authorized users.

## Background

Colorectal cancer (CRC) is the third most common cancer in men and second most common cancer in women worldwide [1, 2]. When tested and screened early, as high as 90% of CRCs could be prevented [3]. Screening has been shown to be cost-effective and ultimately results in decreased CRC incidence and mortality [4]. However, it is estimated that approximately half of individuals diagnosed with CRC will have discovered the cancer at a later stage [3]. This situation emphasizes the importance of prevention and early detection procedures that can interrupt CRC development and progression, especially among populations at higher risk for CRC.

CRC arises from a combination of inherited susceptibility and environmental factors. Several personal factors are related to increased risk of CRC including a history of inflammatory bowel disease, a family history of CRC in a first-degree relative (FHCRC) and previous history of colon or rectum adenomatous polyps [5, 6]. FHCRC is known to increase the risk of CRC, the magnitude of which is dependent on the number of relatives, age of the relative at diagnosis and the degree of relation [7]. The lifetime risk of developing CRC is increased by approximately 100% in those with a first-degree relative diagnosed with CRC [8, 9]. Furthermore, patients diagnosed with low risk adenomas have a higher risk of metachronous advanced neoplasms compared to patients with no adenomas [10].

Excess body weight (being overweight (body mass index (BMI) ≥ 25 kg/m^2^ and <30 kg/m^2^) or obese (BMI ≥ 30 kg/m^2^) has been consistently related to increased risk of CRC. Being overweight or obese can have physiological implications, particularly in the immune and endocrine system, leading to an increase of pro-inflammatory adipokine levels [11]. An overweight BMI can substantially increase the risk of CRC by approximately 9%, and for an obese BMI the risk increase is up to 19%, when compared to those that have a normal BMI [12].

The epidemiologic evidence on the association between physical activity and reduced CRC risk has been classified as “convincing” by the World Cancer Research Fund/American Institute for Cancer Research [13]. Based on observational epidemiological evidence, the reduction in the risk associated with regular physical activity is estimated to be 25–30%, when comparing the most active to least active participants in these studies [14–17]. The effects of physical activity on colorectal tumorigenesis are multifactorial and may be influenced by the parameters of physical activity such as the type, intensity, frequency and duration of activity [18, 19]. It remains to be determined whether or not physical activity provides an equal, or stronger, protective effect amongst “high-risk” populations who are at an increased absolute risk for CRC (i.e. those with a personal or family history of CRC or with particular hereditary syndromes). Depending on regional guidelines, high-risk populations are recommended to undergo augmented screening programs. In this population, the absolute risk for CRC is elevated, which suggests an opportunity for prevention.

Previous meta-analyses have demonstrated that physical activity is associated with a significantly decreased risk of CRC [15, 20]. However, the impact of physical activity in higher risk populations has not yet been established, furthermore whether there is a differential association between high- and low-risk populations has not yet been established. The purpose of this systematic review and meta-analysis is to estimate the relative risk associated with physical activity and CRC risk in higher risk populations, including those with FHCRC, and in overweight and obese populations.

## Methods

### Study selection

Relevant studies were identified through a search of the MEDLINE database using PubMed, conducted through July 11, 2017. We used a number of keywords and medical subject headings indicative of physical activity, CRC and higher risk populations or strong risk factors for CRC (i.e. alcohol, tobacco, first-degree FHCRC, excess BMI, history of polyps, energy intake, etc.) to identify epidemiologic studies investigating the association between physical activity and risk of CRC among subgroups at higher risk. A detailed search strategy is provided in Additional file 1: Table S1. The search was not restricted by date or geographical area. Abstracts, unpublished results, conference proceedings, media articles and studies not published in English were excluded. In addition, reference lists of included articles and previous reviews of physical activity and CRC risk [15, 20, 21] were screened for additional relevant articles.

The initial screening of articles was completed by two independent reviewers (J.D. and R.J.) and updated independently by a third reviewer (C.S.). In cases of discrepancies between reviewers, the senior author (D.B.) was consulted. Predefined study inclusion criteria were: 1) incident CRC as the outcome, 2) exposure of recreational physical activity, total physical activity or transportation-related physical activity, 3) separate effect estimates for subgroups of higher risk individuals, including those with previous FHCRC, previous polyps (adenoma) or those who are overweight or obese (BMI ≥ 25 kg/m^2^). Studies were excluded if: the exposure was limited to only occupational, household or light-intensity activity; the population was limited to those with a previous CRC diagnosis and professional or elite athletes; the outcome was a benign disease or in situ tumor; or the study design was cross-sectional, ecologic, a community-based intervention or a case study.

### Data extraction

Study characteristics and effect estimates were extracted using a standardized abstraction form following the Preferred Reporting Items for Systematic Reviews and Meta-Analyses (PRISMA) guidelines [22]. Data were extracted by one reviewer (R.J. or C.S.) with independent verification by another author (E.S.). For each study, we extracted information on study design, number of cases and controls, assessment of physical activity and CRC, effect estimates and adjustments for confounding, in addition to characteristics of study participants. For the effect estimates, we extracted hazard ratios (HRs), odds ratios (ORs) or relative risk (RRs) with accompanying 95% confidence intervals (CIs) for the risk of CRC associated with the comparison of the most active to the least active group. Depending on how BMI was categorized in some studies, multiple effect estimates were obtained at times and treated as separate populations [23]. The reciprocal value of the effect estimate was taken if physical inactivity was the exposure. In our analyses, we combined effect estimates across study designs where relevant, assuming HRs and ORs as approximations of the relative risk. In studies where subgroup analyses were indicated in the analytic methods but not presented in the article, corresponding authors were contacted via email for the data.

### Subgroup analyses

For BMI subgroups, risk estimates for all subgroups were taken and classified in “low” and “high” BMI groups, depending on how the subgroups were divided in the studies. In general, “low” BMI groups represented those below the median value or the lowest tertile of a study and those in the “normal” range of BMI (<25 kg/m^2^). Effect estimates that were classified as “high” BMI generally represented those above the median BMI or the higher two tertiles of a study and those in the “overweight” (25 ≤ BMI < 30 kg/m^2^) or “obese” (BMI ≥ 30 kg/m^2^) ranges of BMI.

Where there was a sufficient number of studies for a given subgroup analysis (*n* ≥ 3), studies were stratified based on sex (male, female or combined), study design (cohort or case-control), cancer site (colon, rectal or colorectal) and whether or not the effect estimate represented the effects from an interaction of physical activity and BMI on risk of CRC. In instances of an interaction, effect estimates were presented with combined OR/RRs for the lowest BMI and highest physical activity group as the referent category. Studies were also grouped based on physical activity measurements and assessments, including the type of physical activity measured (total, recreational or commuting), timeline of measurement (lifetime or adulthood, past year/two years or unspecified/regular activity), and method of measurement (metabolic equivalent of task (MET)-h/week, kcal/week, number of times/week or month, or other form of measurement).

### Statistical analysis

DerSimonian and Laird random-effects models were used to calculate pooled effect estimates from the included studies [24]. Overall pooled effects in each higher or lower risk subgroup were estimated, as well as stratified by sex, study design, cancer site, analysis of an interaction with BMI, geographical area and the assessment of physical activity (type, time and units of measurement, reference group of physical activity used).

Heterogeneity across studies was assessed using the Cochran’s Q test and the *I*^*2*^ statistic for the overall estimates, as well as the stratified estimates. Substantial statistical heterogeneity was considered to be present if the *p*-value of this statistic was <0.05 and the *I*^*2*^ statistic was greater than 75% [25]. Stratum-specific analyses and meta-regressions were also performed based on stratification by the above-mentioned variables to compare both within and between BMI or FHCRC subgroups. Lastly, the Begg test, visual inspection of funnel plots and Egger’s regression test were used to assess potential publication bias [26]. Additionally, a crude sensitivity analysis was performed to determine if the removal of any one study substantially changed the pooled effect estimate or heterogeneity of the overall analysis. All statistical analyses were performed using STATA® (version 14) and assessed with a 95% significance level, while forest plots were generated using R (version 9.3) [27].

## Results

### Study selection

**Fig. 1:**
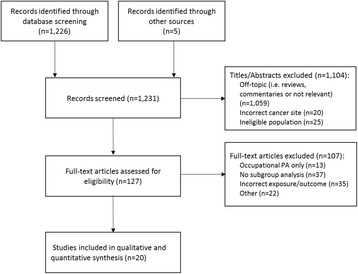
Flow diagram of systematic review and meta-analysis of physical activity and risk of colorectal cancer with higher risk subgroups

The initial search identified 1226 articles and 1231 articles were screened for titles and abstracts, including an additional five articles later identified through manual searches of reference lists (Fig. 1). Of these, 127 articles were further screened and assessed by full-text for inclusion in the systematic review and meta-analysis. Most articles were excluded due to lack of effect estimates stratified by higher risk subgroups, and ultimately, 20 articles covering 18 study populations were included in the meta-analysis [16, 28–46]. Three articles covered the same study population [35–37]; one article was used for the FHCRC subgroup analysis [37], and while two articles contained estimates for the BMI subgroup analysis, [35, 36] only the article containing BMI subgroups by separate sex was used for the meta-analysis [36].

Three of these articles included estimates of the association of physical activity in CRC risk by FHCRC subgroups [28, 32, 37] and 17 articles assessed this association by BMI subgroups [16, 29–31, 33–36, 38–46]. A total of six effect estimates were extracted for associations by FHCRC [28, 32, 37], while 63 effect estimates were extracted for associations by BMI subgroups [16, 29–31, 33–36, 39–44] as some studies reported more than two BMI subgroups [16, 31, 33, 34, 36, 38, 40, 46] or gave separate estimates by sex [31, 33, 36, 39, 43, 44] or cancer site [29, 34]. Additionally, we contacted six authors for additional data related to subgroup analyses of physical activity and colorectal cancer risk, and received the data requested from two studies [29, 34].

### Study characteristics

A summary of the 18 included studies is presented alphabetically by study design in Table 1. There were nine case-control studies (eight population-based and one hospital-based) and nine prospective cohort studies, with three articles covering the same study population [35–37]. All studies were conducted in adult populations, with ages ranging from 18 to 85 years of age. Most studies contained estimates for both sexes, together [16, 28–30, 32, 37, 38, 45, 46] or separate [31, 33, 35, 36, 39, 43, 44], although there were a few studies that consisted of only males [34, 40–42]. Seven studies were conducted in the United States [35–39, 41, 42, 45, 46], five studies were conducted in Europe [15, 16, 28, 30, 40], two in Canada [33, 34], three in Asia [31, 32, 43], and one in Australia [29]. All studies included in this meta-analysis contain at least 100 cases of CRC, although this was not a predefined inclusion criterion. The number of cases ranged from 147 to 4151.

**Table 1:** Study characteristics and quality assessment of studies included in systematic review of physical activity and risk of colorectal cancer with higher risk subgroups

First author, year	Age Range (years)	Country	Case-control: control sample & matching Cohort: study name		Physical activity period	Comparison of Physical Activity	Cancer Site/Number of Cases	Method of Case Confirmation	Assessment of Confounding	Subgroups analyzed	Method of Subgroup Assessment
Case-Control Studies											
Boutron-Ruault, 2001 [28]	30–79	France	Population	No matching	Regular^a, b^	Sedentary vs. High (regular physical exercise)	Colorectal: 171	Histological exam	Age and sex	FHCRC	Interview questionnaire
Boyle, 2012 [29]	40–79	Australia	Population	Frequency	Adulthood (≥19 years)	Definite vs. Never Resistance training	Colon: 552 Rectal: 318	Histological exam	Age group, sex, resistance training in other age periods, lifetime moderate and vigorous non-resistance training rerecraetional physical activity, lifetime occupational activity and energy intake	BMI	Self-administered questionnaire
Gerhardsson de Verdier, 1990 [30]	40–69	Sweden	Population	Frequency	1950-1985^b^	Very active vs. fairly active	Left colon: 147	Histo-pathological diagnosis and ICD-7 codes	Year of birth and sex	BMI	Self-administered questionnaire
Hou, 2004 [31]	30–74	China	Population	Frequency	Lifetime	>94.3 MET-h/wk. vs. <48.3 MET-h/wk	Colon: 931	Histo-pathology or other methods	Age, education, family income, marital status, total energy intake, intake of red meat, carotene and fiber, number of pregnancies and menopausal status	BMI	Interview questionnaire
Huang, 2004 [32]	≥18	Japan	Non-cancer outpatients	No matching	Regular^a^	≥3 times/month vs. <3 times/month	Colorectal: 1352	Medical data from the Aichi Cancer Center Hospital	Age, sex	FHCRC	Interview questionnaire
Mao, 2003 [33]	20–76	Canada	Population	Frequency	Past 2 years	≥24.6 MET-h/wk. vs. <8.8 MET-h/wk	Rectal: 1447	Histological exam	Age, province, education, total caloric dietary fiber, vegetable and fruit intake, smoking and alcohol consumption	BMI	Self-administered questionnaire
Parent, 2011 [34]	35–70	Canada	Population	Frequency	Overall activity in adult life^b^	Higher (active at work with recreational PA or very active at work) vs. Lower (sedentary at work or active at work without recreational PA)	Colon: 496 Rectal: 248	Pathological confirmation	Age, socio-economic status, education, ethnicity, respondent status, smoking, alcohol/β-carotene (colon) and beer (rectum)	BMI	Interview questionnaire
Slattery, 1997 [35–37]	30–79	USA	Population	Frequency	Lifetime	High (>1000 kcal/wk) vs. Low (0 kcal/wk)	Colon: 2073	Pathology report	Age at diagnosis, FHCRC, BMI, dietary fiber and calcium, use of aspirin or NSAIDs, dietary cholesterol and energy intake	BMI and FHCRC	Interview questionnaire
Zhang, 2006 [38]	40–85	USA	Population	Frequency	Adulthood	≥2 times/wk. vs. <1 time/month	Colon: 685	Histological exam	Age, sex, education level, dietary intake of fat and fiber and FHCRC	BMI	Self-administered questionnaire
Cohort Studies											
Ballard-Barbash, 1990 [39]	30–62	USA	Framingham Study		Regular^a^	High vs. low tertile of PA index	Colon: 152	Medical history from cohort records	Age	BMI	Interview questionnaire
Friedenreich, 2006 [16]	35–70	Europe	European Prospective Investigation into Cancer and Nutrition		Past year	Active vs. Inactive	Colon: 1094	Population based cancer registries, health insurance records, pathology registries	Age, centre, energy and fibre intake, education, smoking	BMI	Self-report and clinical examination
Larsson, 2006 [40]	45–79	Sweden	Cohort of Swedish Men (COSM)		Year before study enrolment	≥60 min/day vs. <10 min/day	Colorectal: 496	ICD-9 codes in the National and Regional Swedish Cancer registers	Education, FHCRC, history of diabetes, smoking and aspirin use	BMI	Self-administered questionnaire
Lee, I., 1994 [41]	30–79	USA	Harvard Alumni Health Study		Regular^a^	Highly active (≥2500 kcal/wk) vs. Inactive (<1000 kcal/wk)	Colon: 280	Questionnaire and death certificates	Age, BMI and parental history of CRC	BMI	Self-administered questionnaire
Lee, I., 1997 [42]	40–84	USA	Physician’s Health Study		Regular^a^	1+ times/week vs. <1 time/week	Colon: 217	Pathology reports in medical records	Age, alcohol consumption and treatment assignment	BMI	Self-administered questionnaire
Lee, K., 2007 [43]	40–69	Japan	Japan Public Health Center-Based Prospective Study (JPHC)		Regular^a,b^	>43.75 MET-h/day vs. <28.25 MET-h/day	Colorectal: 486	Population-based cancer registries and death certificates, 94.7% verified by histological exam	Age, study area, FHCRC, smoking, alcohol intake, intake of red mean, dietary fiber and folate	BMI	Self-administered questionnaire
Morikawa, 2013 [45]	30–75	USA	Health Professionals Follow-up Study (HPFS) and the Nurses’ Health Study (NHS)		Regular (mean of all past 2-year measures)	≥9 MET-h/wk. vs. <9 MET-h/wk.	Colorectal: 861	Medical records and pathology reports	Alcohol, folate, vitamin D, calcium, caloric and red meat intake, current smoking status, smoking before 30 years of age, current multivitamin use, current aspirin use, previous sigmoidoscopy, and family history of colorectal cancer	BMI	Self-administered questionnaire
Schmid, 2016 [46]	50–71	USA	National Institutes of Health AARP Diet and Health Study (NIH-AARP Diet and Health Study)		Past 10 years^b^	>7 h/wk. MVPA vs. None	Colon: 4151	Cancer registry records. Self-reports and confirmation through medical records used to ascertain completeness of reporting (~90%)	Age, sex, race/ethnicity, education, smoking status, family history of colon cancer, hormone replacement therapy use (women only), nonsteroidal anti-inflammatory drug use, total fiber intake, red meat intake and alcohol consumption	BMI	Self-administered questionnaire
Thune, 1996 [44]	20–49	Norway	Population-based Cohort of Norway		Past year	Active vs. sedentary	Colon: 335	Cancer Registry of Norway, 95% verified by histological exam	Age at entry, geographic region	BMI	Self-administered questionnaire with clinical examination for inconsistency

*Abbreviations: BMI* body mass index, *FHCRC* first-degree family history of colorectal cancer, *MET* metabolic equivalent of task, *hr./wk.* hours per week, *PA* physical activity, *CRC* colorectal cancer

^a^Time period of physical activity assessed not specified

^b^Measures overall activity (occupational and recreational)

Given that the outcome of interest is incidence of CRC, most studies included used histopathological exams as a method of case confirmation, with the exception of five studies [32, 39–41, 46], that relied on registries, medical records or death certificates. In terms of physical activity measurements, all were self-reported measures of physical activity. Questionnaires were either self-administered [16, 28–30, 33, 38, 40–46] or administered by a trained interviewer [31, 32, 34–37, 39]. In terms of the time period of measurement, only six studies assessed lifetime physical activity [29–31, 34–38], which would be considered our ideal period of measurement to determine disease etiology and possible associations. Two studies had a relatively long period of physical activity measurement (10–17 years) [45, 46], four studies assessed the past year or two years prior to the questionnaire or interview [16, 33, 40, 44] and six studies had an undefined period of physical activity assessment [28, 32, 39, 41–43]. The Newcastle-Ottawa Scale was used to assess the quality of each study and is summarized in Additional file 1: Table S2.

### Meta-analysis

#### FHCRC subgroups

**Fig. 2:**
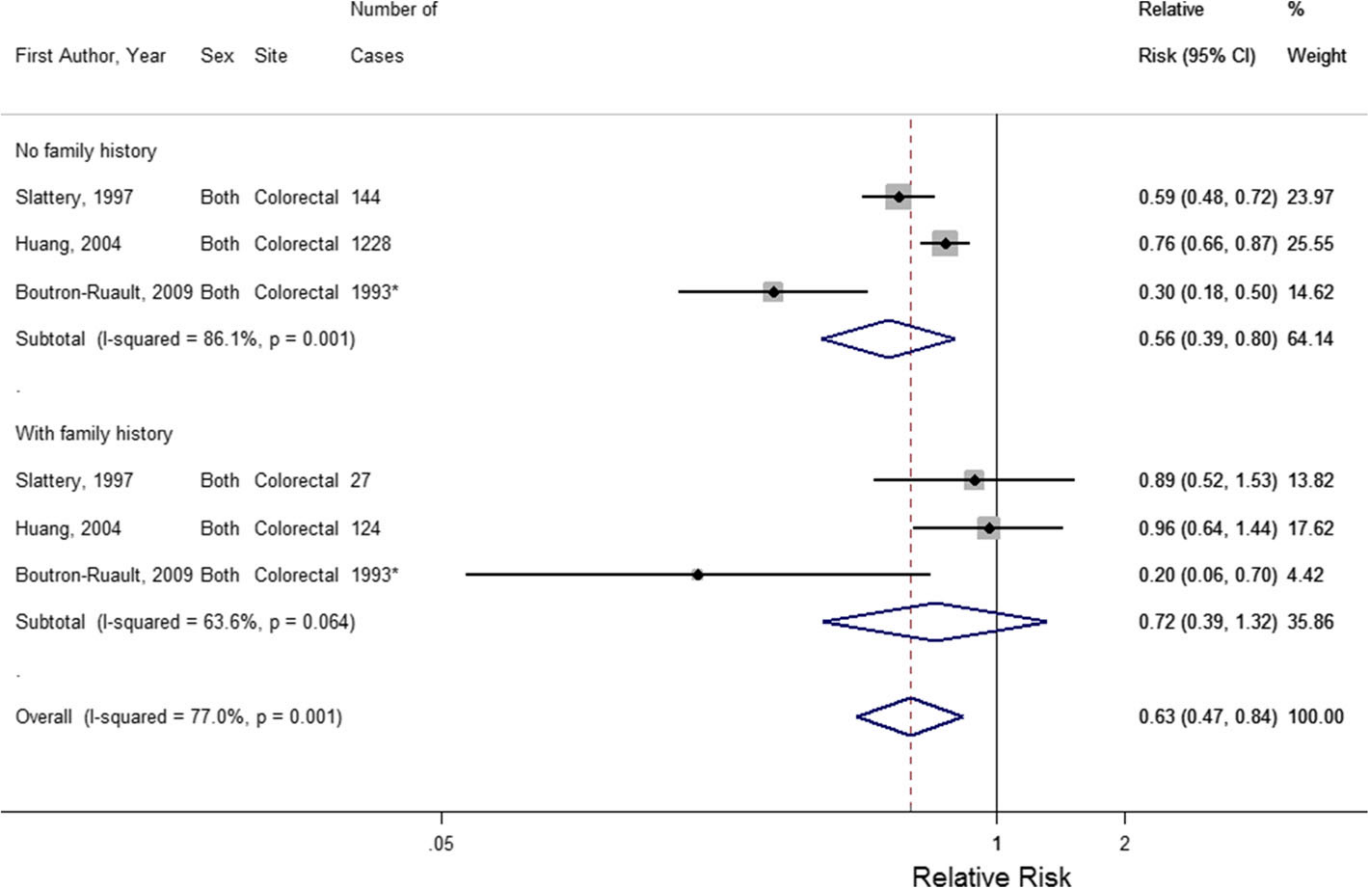
Adjusted relative risk estimates of physical activity and colorectal cancer risk stratified by family history, listed in chronological order (p-value across subgroups = 0.586). All estimates are for both sexes. * Family history subgroup-specific case numbers were not described for Slattery (1997) [37], thus total cases were used for case numbers from this study

In the meta-analysis of studies including FHCRC subgroups, the overall relative risk of CRC associated with physical activity was 0.56 (95% CI: 0.39–0.80) in those without FHCRC, while it was 0.72 (95% CI: 0.39–1.32) in those with FHCRC (Fig. 2). While the pooled estimate in those without FHCRC was statistically significantly associated with a decreased risk of CRC, there was no difference between pooled estimates in those with or without FHCRC, as the *p*-value for the between group comparison was 0.586.

#### BMI subgroups

**Fig. 3:**
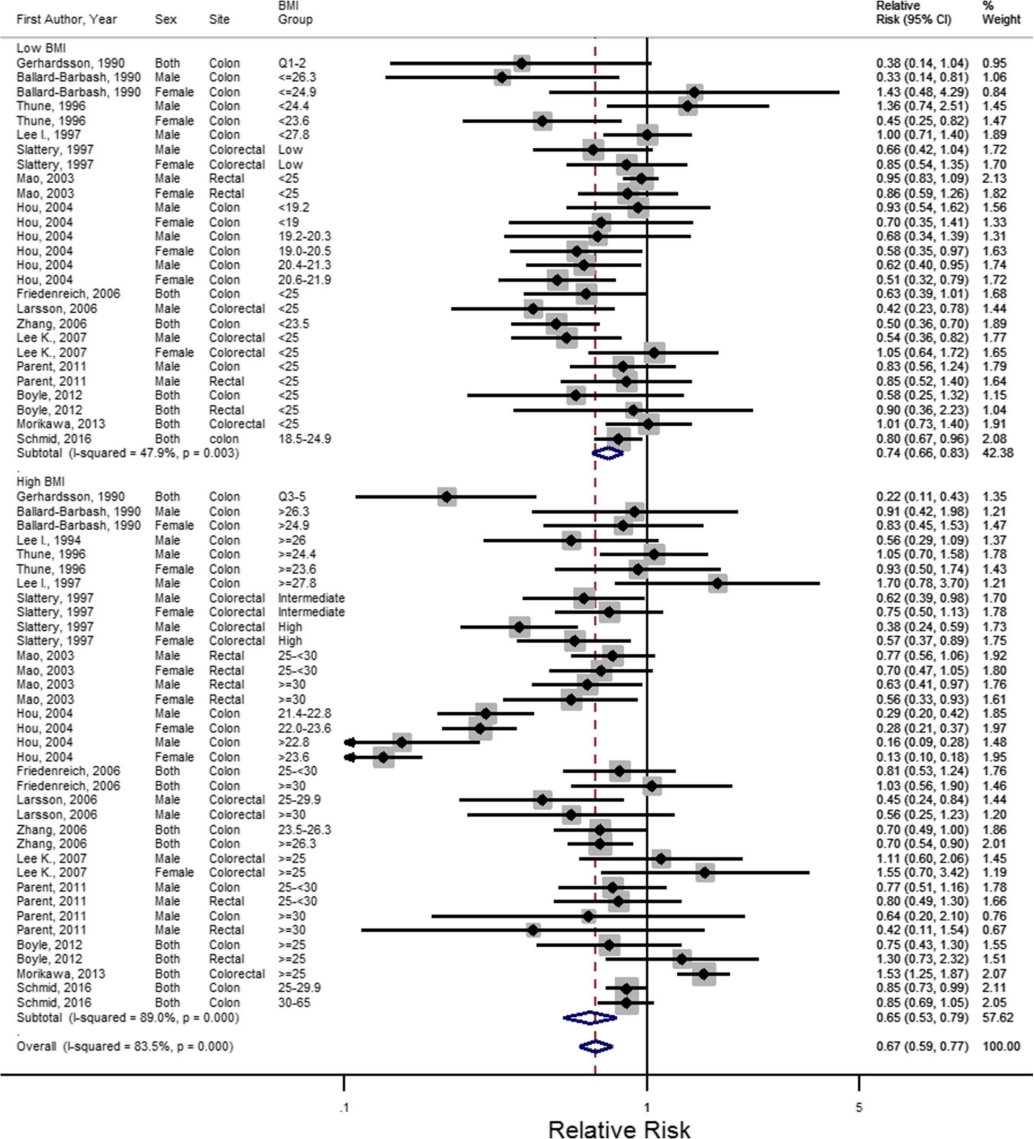
Adjusted relative risk estimates of physical activity and colorectal cancer risk stratified by BMI subgroups, listed in chronological order

In the analysis of BMI subgroups, the pooled estimate for the relative risk of CRC associated with physical activity was 0.74 (95% CI: 0.66–0.83) in the lower BMI group and 0.65 (95% CI: 0.53–0.79) in the higher BMI group (Fig. 3, stratified by study design). In both BMI groups, physical activity was significantly associated with a decreased risk of CRC, although the difference between groups was not significant (*p* = 0.389) in the overall analysis (Table 2).

**Table 2:** Overall and stratified meta-analyses of relative risk estimates (associations) for physical activity and risk of colorectal cancer by BMI subgroups

Overall/stratified analysis	Total number of estimates	Number of cases^a^	Pooled RR (95% CI)	*I*^*2*^ (%)	P_heterogeneity_	P across subgroups	P across high and low BMI
Low BMI							
Overall	27	7045	0.74(0.66–0.83)	47.9	0.003		
Sex							
Male	12	2590	0.76 (0.64–0.92)	53.1	0.015	0.551	
Female	8	1639	0.72 (0.57–0.91)	33.6	0.160		
Both	7	2816	0.70 (0.56–0.89)	50.2	0.061		
Study Design							
Cohort	11	3073	0.76 (0.61–0.94)	59.3	0.006	0.088	
Case-control	16	3972	0.72 (0.62–0.84)	40.7	0.046		
Cancer Site							
Colon	17	3436	0.69 (0.59–0.81)	41.5	0.038	0.410	
Colorectal	6	2854	0.74 (0.56–0.98)	55.6	0.047		
Rectal	4	755	0.94 (0.83–1.06)	0.0	0.942		
Interaction^b^							
No	13	3128	0.78 (0.66–0.92)	37.3	0.004	0.462	
Yes	14	3917	0.70 (0.59–0.84)	57.6	0.085		
Physical Activity Type							
Total	9	2681	0.74 (0.61–0.89)	32.7	0.156	0.605	
Recreational	12	3874	0.78 (0.64–0.94)	60.5	0.003		
Commuting	6	490	0.64 (0.51–0.79)	0.0	0.675		
Physical Activity Measurement							
Adulthood/lifetime^c^	16	5151	0.72 (0.64–0.82)	16.0	0.271	0.497	
Past year or two	6	1319	0.75 (0.56–1.01)	67.8	0.008		
Regular^d^	5	575	0.77 (0.51–1.17)	63.8	0.026		
Physical Activity Assessment							
MET-h/day or week	13	2188	0.73 (0.62–0.87)	52.4	0.014	0.969	
Kcal/week	2	1993	0.75 (0.54–1.04)	0.0	0.437		
Times/week or month	3	1939	0.75 (0.53–1.05)	78.1	0.010		
Other^e^	9	925	0.72 (0.54–0.98)	41.6	0.090		
Physical Activity Reference Group							
No activity	9	4453	0.70 (0.57–0.85)	41.9	0.088	0.538	
Some activity	18	2592	0.76 (0.65–0.88)	47.8	0.013		
Geographical Region							
North America	12	5026	0.82 (0.71–0.94)	47.7	0.033	0.122	
Europe	5	920	0.60 (0.38–0.93)	59.2	0.044		
Asia	8	824	0.66 (0.55–0.80)	7.4	0.373		
Australia	2	275	0.71 (0.38–1.30)	0.0	0.482		
High BMI							
Overall	36	9407	0.65 (0.53–0.79)	89.0	<0.001		0.389
Sex							
Male	17	2928	0.56 (0.34–0.94)	74.9	<0.001	0.268	0.264
Female	9	1568	0.61 (0.47–0.79)	92.1	<0.001		0.333
Both	10	4911	0.83 (0.65–1.05)	82.8	<0.001		0.384
Study Design							
Cohort	15	4625	0.93 (0.78–1.13)	64.1	<0.001	0.002	0.178
Case-control	21	4782	0.51 (0.39–0.66)	88.0	<0.001		0.053
Cancer Site							
Colon	20	5330	0.58 (0.44–0.79)	91.5	<0.001	0.282	0.435
Colorectal	9	2841	0.74 (0.49–1.11)	85.9	<0.001		0.948
Rectal	7	1236	0.74 (0.62–0.88)	4.1	0.395		0.052
Interaction^b^							
No	18	4881	0.87 (0.79–0.96)	0.0	0.761	0.001	0.220
Yes	18	4526	0.48 (0.34–0.68)	93.4	<0.001		0.081
Physical Activity Type							
Total	13	4141	0.81 (0.68–0.96)	40.4	0.065	<0.001	0.424
Recreational	19	4830	0.74 (0.61–0.91)	75.3	<0.001		0.810
Commuting	4	436	0.21 (0.14–0.32)	81.9	0.001		0.001
Physical Activity Measurement							
Adulthood/lifetime^c^	20	7082	0.54 (0.40–0.73)	93.5	<0.001	0.022	0.179
Past year or two	10	1985	0.75 (0.64–0.87)	3.5	0.408		0.780
Regular^d^	6	340	1.00 (0.73–1.38)	21.7	0.270		0.366
Physical Activity Assessment							
MET-h/day or week	13	2411	0.55 (0.37–0.81)	90.8	<0.001	0.086	0.224
Kcal/week	5	2058	0.57 (0.45–0.72)	20.3	0.285		0.251
Times/week or month	5	3549	0.90 (0.67–1.19)	87.9	<0.001		0.478
Other^e^	13	1389	0.77 (0.59–1.00)	49.8	0.030		0.778
Physical Activity Reference Group							
No activity	14	8521	0.77 (0.67–0.87)	41.2	<0.001	0.132	0.474
Some activity	22	4745	0.57 (0.40–0.82)	92.4	0.054		0.199
Geographical Region							
North America	21	6990	0.75 (0.64–0.87)	69.6	<0.001	0.127	0.525
Europe	7	1239	0.67 (0.45–0.99)	69.9	0.003		0.724
Asia	6	583	0.36 (0.19–0.66)	92.0	<0.001		0.090
Australia	2	595	0.98 (0.57–1.68)	45.1	0.177		0.520

*Abbreviations*: *BMI* body mass index, *MET* metabolic equivalent of task

^a^BMI subgroup-specific case numbers were not described for Gerhardsson (1990) [30] Slattery (1997) [36], thus total cases were used for case numbers from these two studies

^b^Estimates representing an interaction between physical activity and BMI on colorectal cancer risk

^c^Studies with over 10 years of physical activity measurement were included in this category

^d^Time period of measurement not specified

^e^Other measures include a study’s own physical activity index or unique classification of active vs. inactive individuals

In further analyses, we stratified our estimates by age, cancer site, study design, whether or not the comparison was for an interaction of BMI and physical activity [30, 31, 33, 36, 38, 40, 45], as well as the different aspects of physical activity assessments in each study and geographical region (Table 2). Within the low BMI group, there were no statistically significant differences across subgroups of the stratified analysis as all *p*-values were non-significant. However, in the high BMI group, we did observe that there was a statistically significant difference in estimates based on study design (*p* = 0.002), presence of an interaction between physical activity and BMI (*p* = 0.001), as well as methods of assessing physical activity in terms of type (*p* < 0.001) and time period of measurement (*p* = 0.022). Additionally, we observed several strongly protective associations in the high BMI group in the stratified analyses, particularly in case-control studies (pooled RR = 0.51, 95% CI: 0.39–0.66) and in the timing of physical activity assessment with adulthood/lifetime measurement showing a very strong protective association (pooled RR = 0.54, 95% CI: 0.40–0.73). When comparing across BMI subgroups, there were generally no significant differences between low and high BMI relative risks, with the exception of case-control studies (*p* = 0.053) and studies of rectal cancer (*p* = 0.052), which bordered on significance, in addition to the study by Hou et al. [31] that assessed commuting physical activity (p = 0.001).

### Heterogeneity

In the analysis of FHCRC subgroups, there was considerable heterogeneity between these three studies in estimates in those without FHCRC (*I*^*2*^ = 86.1%, P_heterogeneity_ = 0.001) and in those with FHCRC (*I*^*2*^ = 63.6%, P_heterogeneity_ = 0.064). Given the small sample size of only three studies, we could not investigate the source of this heterogeneity.

Similar to the FHCRC subgroup estimates, there was a high degree of heterogeneity between studies in both the lower BMI group (*I*^*2*^ = 47.9%, P_heterogeneity_ = 0.003, *n* = 27 estimates from 15 studies) and the higher BMI group (*I*^*2*^ = 89.0%, P_heterogeneity_ < 0.001, *n* = 36 estimates from 16 studies). In our stratified analysis, we found that the variables used to stratify did not explain a majority of the heterogeneity in the low BMI subgroup, as all *p*-values across subgroups were greater than 0.05. In the high BMI subgroup, we observed that study design, presence of an interaction with BMI and the measurement of physical activity (type and period of measurement) likely played a role in the heterogeneity of estimates, as they all had p-values less than 0.05.

### Publication bias

**Fig. 4:**
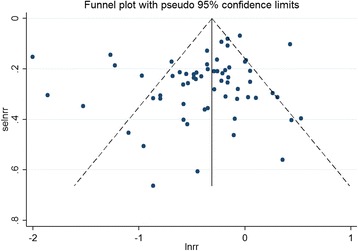
Funnel plot of study estimates of colorectal cancer risk with physical activity by high and low BMI subgroups

A funnel plot was generated to assess the presence of publication bias in the included studies showed a fairly symmetrical distribution of effect estimates (Fig. 4). While there was some visual asymmetry present in the funnel plot, Begg’s test for small study effects and Egger’s regression test found no evidence of publication bias in the overall number of studies (*p* = 0.352 and *p* = 0.077, respectively). These tests may, however, have been limited in their statistical power by the small number of included estimates. We did not conduct a publication bias test for studies examining family history because of the small number of studies included with FHCRC subgroups.

### Sensitivity analysis

A sensitivity analysis wherein studies were individually removed from the meta-analyses was performed for BMI subgroups, but not FHCRC subgroups due to the small number of studies identified. In this analysis, we did not observe any substantial changes in the heterogeneity of the studies, with the removal of any one study (Additional file 1: Table S3). All *p*-values for heterogeneity tests were still statistically significant. With respect to pooled effect estimates, one study by Hou et al. [31] was found to influence the effect estimate of the high BMI subgroup considerably (Additional file 1: Table S3). Furthermore, we completed a sensitivity analysis to investigate the effects of normal (BMI < 25 kg/m^2^), overweight (25 ≤ BMI < 30 kg/m^2^) and obese BMI (BMI ≥ 30 kg/m^2^), as classified by the World Health Organization. There were five studies that reported BMI using this criteria [16, 33, 34, 40, 46], and the analysis revealed no statistically significant differences in the effect estimates for the association of physical activity and colorectal cancer risk across subgroups of BMI (*p* = 0.29, data not shown).

## Discussion

In this meta-analysis, the differential associations between physical activity and the relative risk of CRC by the presence of a FHCRC and BMI subgroups were explored. We did not observe that FHCRC significantly modifies the association between physical activity and the relative risk of CRC. Additionally, while a stronger protective association between physical activity and CRC risk was observed in the high BMI group, the difference in the overall pooled risk estimates between the low and high BMI subgroups was not statistically significant. Our literature search identified nine case-control and nine cohort studies that investigated the association between physical activity and risk of CRC across higher risk subgroups. To our knowledge, no experimental studies have been conducted for this association due to the size and time period of study that would be required to have CRC incidence as an outcome.

Based on our literature search, only three studies contained effect estimates stratified by the presence of a FHCRC. Physical activity was significantly protective for CRC risk in those without FHCRC, while this association was not statistically significant in those with FHCRC. However, the risk was not statistically significantly different between groups. Because of this small sample size, we were not able to explore the effect of FHCRC on the relation between physical activity and relative risk of CRC further, despite the strong heterogeneity between studies. Since family history is often a proxy for genetic susceptibility, we initially hypothesized those with FHCRC would have a more pronounced protective effect of physical activity on CRC risk. In this meta-analysis, we did not observe a statistically significant difference between subgroups and there was a slightly stronger effect in those without FHCRC. This effect was likely because of the large study by Huang et al., [32] which found a statistically significant difference between subgroups of FHCRC, with a statistically significant protective effect of physical activity in those without FHCRC. Additionally, it is possible that while FHCRC should increase risk of CRC, this increased risk is attenuated by increased physical activity and thus, physical activity can prove to be an effective lifestyle modification in cancer prevention for those with FHCRC. However, this meta-analysis was underpowered to determine a conclusive effect of FHCRC on the association between physical activity and risk of CRC.

With respect to BMI subgroups, we found that there was a stronger protective association between physical activity and CRC risk in the higher BMI group, which was in agreement with our hypothesis that higher risk groups can have a further reduced risk of CRC with physical activity. Borderline statistically significant associations across BMI subgroups were seen in risk estimates of only rectal cancer and in case-control studies. There were only three studies that provided separate effect estimates for rectal cancer [29, 33, 34], however, one study was particularly large, with 1447 cases of rectal cancer [33], potentially providing more power to these estimates. While a previous meta-analysis of the association between BMI and cancer incidence of various sites found that BMI had less of an effect on incidence of rectal cancer compared to colon cancer [47], the present meta-analysis indicates that the association between physical activity and CRC risk is substantially more beneficial in those with higher BMI. In our stratified analyses of BMI subgroups, we also observed a substantially lower risk of CRC with physical activity with higher BMI in the seven case-control studies identified in our search. We hypothesize that this difference could be attributable to biases associated with case control studies, such as recall bias or selection bias, which may overestimate the true association of physical activity and risk of colorectal cancer. In the absence of recall or selection bias, it is possible that case-control studies had more detailed measurement of physical activity in their questionnaires, which could have resulted in less measurement error and therefore, less attenuation of the relative risks.

Due to the inclusion of multiple effect estimates from some studies compared with other studies with only one estimate, we performed a sensitivity analysis with only one effect estimate from each study to confirm that the effect estimates from one study were not overly influencing others. We did not observe any substantial impact due to potential non-independence of results in any of our analyses (data not shown).

Previous studies have shown stronger protective effects of increased lifetime or adulthood physical activity with not only CRC, but other cancer sites as well, and heterogeneity across studies is largely attributable to characterization of physical activity [48, 49]. Much of the heterogeneity observed in this meta-analysis could also be attributed to differences in measurement of physical activity as the type, time period measured, reference group and quantification of physical activity were significantly different across these studies. While the differences in these variables are only statistically significant in the high BMI group, there was a clear trend across both BMI subgroups showing that measurement of adulthood or lifetime physical activity was associated with the most beneficial effect on the risk of CRC. This finding supports previous studies that have shown stronger protective effects of increased lifetime or adulthood physical activity with not only CRC, but other cancer sites as well [48, 49]. However, few studies have investigated the role of the timing of physical activity exposure in life and further research in this area is necessary [48] While.

In a sensitivity analysis in which studies were individually removed from the BMI subgroup analysis, no study was found to impact the heterogeneity of studies in the analysis significantly. However, the study by Hou et al. [31] was found to contribute substantially to a more protective association of physical activity in relation to risk of CRC. This study was the only investigation in our analysis that measured commuting physical activity, which can represent a large amount of activity and contribute considerably to the protective association observed. In addition, there were two studies with small sample sizes [28, 39], which may have contributed to the heterogeneity between the studies since the small sample sizes from these two studies are reflected in the precision of their effect estimates.

In addition to the small sample size of studies stratifying by risk factors of CRC, this meta-analysis faced further limitations, common in performing meta-analyses, such as selection bias and the large degree of heterogeneity between studies. Because we limited the search to studies investigating the association of physical activity and colorectal cancer risk that stratified by higher risk subgroups, we likely excluded a number of studies that may have collected this information, but did not report stratified results. Although we did attempt to contact authors, it is possible that selection bias in the inclusion of studies may have occurred. While we did not observe any publication bias in studies that stratified by BMI, we were unable to perform this analysis on studies that stratified by FHCRC due to the low number of studies identified in our search. Furthermore, we observed a large degree of heterogeneity between studies included in this meta-analysis that could be attributed to differences in study design, presence of an interaction with BMI and the measurement of physical activity in each study.

While the mechanisms by which physical activity decreases risk of CRC remain unclear, it is possible that this relation can be modified by BMI, as previously described [35]. It has been hypothesized that physical activity can shorten gastrointestinal transit time, enhance immune function and alter bile acid secretion, serum cholesterol or hormones of the gastrointestinal tract and pancreas [50, 51]. However, most evidence regarding the modification of the association between physical activity and CRC risk by BMI points to changes in insulin sensitivity as the predominant mechanism. Physical activity has been shown to increase insulin sensitivity [52], while obesity decreases insulin sensitivity [53] and it is possible that the interaction of the two can result in more benefit from increased physical activity for high BMI subgroups with respect to CRC risk reduction. Lastly, high-risk populations are under increased surveillance and screening because of an increased absolute risk of cancer, even with the same relative risk, thus, efforts to increase physical activity in these populations may have a greater impact in reducing the cancer burden.

In this meta-analysis, we did not find any studies that examined the impact of physical activity across groups of individuals who have a history of previous colon polyps, or those with strong risk factors for CRC, such as tobacco smoking and alcohol consumption. Additional research in these populations, particularly those with previous adenomas, is warranted to examine the potential for prevention of subsequent CRC. Due to the limited number of studies measuring the effect of physical activity across FHCRC subgroups, there is also a need for additional studies among those with FHCRC in order to better assess how a modifiable lifestyle factor, such as physical activity, can further reduce the risk of CRC in high-risk and high BMI populations [54]. Furthermore, we did not come across enough studies that specifically measured the type, frequency, intensity and durations of physical activity to perform a meta-analysis on these parameters, which are likely important in colorectal tumourigenesis. Thus, additional research on these factors in physical activity is warranted.

## Conclusions

This meta-analysis found a statistically significant overall protective association between physical activity and the risk of CRC, with no statistically significant differences by FHCRC or BMI subgroups. The protective association with physical activity was stronger in the higher BMI subgroup. Increased physical activity could potentially have an added benefit as a method of cancer prevention in higher risk subgroups and can be promoted in screening programs for the higher risk populations.

## Additional file

Additional file 1: Tables S1-S3. PubMed search strategy for systematic review, Newcastle-Ottawa Scale for study quality assessment, and sensitivity analysis for individual study removal. (DOCX 21 kb)

## Abbreviations

BMI: Body mass index
CI: Confidence interval
CRC: Colorectal cancer
FHCRC: First-degree family history of colorectal cancer
HR: Hazard ratio
MET: Metabolic equivalent of task
OR: Odds ratio
PA: Physical activity
PRISMA: Preferred Reporting Items for Systematic Reviews and Meta-Analyses
RR: Relative risk

## References

[1] Ferlay J, Soerjomataram I, Ervik M, Dikshit R, Eser S, Mathers C (2013). GLOBOCAN 2012 v1.0, cancer incidence and mortality worldwide: IARC CancerBase no. 11 [internet].

[2] Torre LA, Bray F, Siegel RL, Ferlay J, Lortet-Tieulent J, Jemal A (2015). Global cancer statistics, 2012. CA Cancer J Clin.

[3] Canadian Cancer Society's Advisory Committee on Cancer Statistics (2015). Canadian Cancer Statistics 2015.

[4] Sharaf RN, Ladabaum U (2013). Comparative effectiveness and cost-effectiveness of screening colonoscopy vs. sigmoidoscopy and alternative strategies. Am J Gastroenterol.

[5] Haggar FA, Boushey RP (2009). Colorectal cancer epidemiology: incidence, mortality, survival, and risk factors. Clin Colon Rectal Surg.

[6] Edwards BK, Ward E, Kohler BA, Eheman C, Zauber AG, Anderson RN (2010). Annual report to the nation on the status of cancer, 1975-2006, featuring colorectal cancer trends and impact of interventions (risk factors, screening, and treatment) to reduce future rates. Cancer.

[7] Schoen RE, Razzak A, KJ Y, Berndt SI, Firl K, Riley TL, et al. Incidence and mortality of colorectal cancer in individuals with a family history of colorectal cancer. Gastroenterology. 2015; 10.1053/j.gastro.2015.07.055 PMC4628587 26255045

[8] Butterworth AS, Higgins JP, Pharoah P (2006). Relative and absolute risk of colorectal cancer for individuals with a family history: a meta-analysis. Eur J Cancer.

[9] Taylor DP, Burt RW, Williams MS, Haug PJ, Cannon-Albright LA (2010). Population-based family history-specific risks for colorectal cancer: a constellation approach. Gastroenterology.

[10] Hassan C, Gimeno-Garcia A, Kalager M, Spada C, Zullo A, Costamagna G (2014). Systematic review with meta-analysis: the incidence of advanced neoplasia after polypectomy in patients with and without low-risk adenomas. Aliment Pharmacol Ther.

[11] Tandon K, Imam M, Ismail BE, Castro F (2015). Body mass index and colon cancer screening: the road ahead. World J Gastroenterol.

[12] Moghaddam AA, Woodward M, Huxley R (2007). Obesity and risk of colorectal cancer: a meta-analysis of 31 studies with 70,000 events. Cancer Epidemiol Biomark Prev.

[13] World Cancer Research Fund and American Institute for Cancer Res Food (2007). Nutrition, physical activity, and the prevention of cancer: a global perspective.

[14] Wolin KY, Glynn RJ, Colditz GA, Lee IM, Kawachi I (2007). Long-term physical activity patterns and health-related quality of life in U.S. women. Am J Prev Med.

[15] Wolin KY, Yan Y, Colditz GA, Lee IM (2009). Physical activity and colon cancer prevention: a meta-analysis. Br J Cancer.

[16] Friedenreich C, Norat T, Steindorf K, Boutron-Ruault MC, Pischon T, Mazuir M (2006). Physical activity and risk of colon and rectal cancers: the European prospective investigation into cancer and nutrition. Cancer Epidemiol Biomark Prev.

[17] Chao A, Connell CJ, Jacobs EJ, McCullough ML, Patel AV, Calle EE (2004). Amount, type, and timing of recreational physical activity in relation to colon and rectal cancer in older adults: the cancer prevention study II nutrition cohort. Cancer Epidemiol Biomark Prev.

[18] McTiernan A (2008). Mechanisms linking physical activity with cancer. Nat Rev Cancer.

[19] Brown JC, Winters-Stone K, Lee A, Schmitz KH (2012). Cancer, physical activity, and exercise. Compr Physiol.

[20] Boyle T, Keegel T, Bull F, Heyworth J, Fritschi L (2012). Physical activity and risks of proximal and distal colon cancers: a systematic review and meta-analysis. J Natl Cancer Inst.

[21] Robsahm TE, Aagnes B, Hjartaker A, Langseth H, Bray FI, Larsen IK (2013). Body mass index, physical activity, and colorectal cancer by anatomical subsites: a systematic review and meta-analysis of cohort studies. Eur J Cancer Prev.

[22] Moher D, Liberati A, Tetzlaff J, Altman DG (2009). The PG. preferred reporting items for systematic reviews and meta-analyses: the PRISMA statement. PLoS Med.

[23] Borenstein M, Hedges LV, Higgins JPT, Rothstein HR. Introduction to Meta-Analysis: John Wiley & Sons, Ltd; 2009. p. 103–212.

[24] DerSimonian R, Kacker R (2007). Random-effects model for meta-analysis of clinical trials: an update. Contemp Clin Trials.

[25] Higgins JP, Thompson SG, Deeks JJ, Altman DG (2003). Measuring inconsistency in meta-analyses. BMJ.

[26] Begg CB, Mazumdar M (1994). Operating characteristics of a rank correlation test for publication bias. Biometrics.

[27] Team RDC (2011). R: a language and environment for statistical computing.

[28] Boutron-Ruault MC, Senesse P, Meance S, Belghiti C, Faivre J (2001). Energy intake, body mass index, physical activity, and the colorectal adenoma-carcinoma sequence. Nutr Cancer.

[29] Boyle T, Bull F, Fritschi L, Heyworth J (2012). Resistance training and the risk of colon and rectal cancers. Cancer Causes Control.

[30] Gerhardsson de Verdier M, Steineck G, Hagman U, Rieger A, Norell SE (1990). Physical activity and colon cancer: a case-referent study in Stockholm. Int J Cancer.

[31] Hou L, Ji BT, Blair A, Dai Q, Gao YT, Chow WH (2004). Commuting physical activity and risk of colon cancer in shanghai, China. Am J Epidemiol.

[32] Huang XE, Hirose K, Wakai K, Matsuo K, Ito H, Xiang J (2004). Comparison of lifestyle risk factors by family history for gastric, breast, lung and colorectal cancer. Asian Pac J Cancer Prev.

[33] Mao Y, Pan S, Wen SW, Johnson KC (2003). Physical inactivity, energy intake, obesity and the risk of rectal cancer in Canada. Int J Cancer.

[34] Parent ME, Rousseau MC, El-Zein M, Latreille B, Desy M, Siemiatycki J (2011). Occupational and recreational physical activity during adult life and the risk of cancer among men. Cancer Epidemiol.

[35] Slattery ML, Potter JD (2002). Physical activity and colon cancer: confounding or interaction?. Med Sci Sports Exerc.

[36] Slattery ML, Potter J, Caan B, Edwards S, Coates A, Ma KN (1997). Energy balance and colon cancer--beyond physical activity. Cancer Res.

[37] Slattery ML, Edwards SL, Ma KN, Friedman GD, Potter JD (1997). Physical activity and colon cancer: a public health perspective. Ann Epidemiol.

[38] Zhang Y, Cantor KP, Dosemeci M, Lynch CF, Zhu Y, Zheng T (2006). Occupational and leisure-time physical activity and risk of colon cancer by subsite. J Occup Environ Med.

[39] Ballard-Barbash R, Schatzkin A, Albanes D, Schiffman MH, Kreger BE, Kannel WB (1990). Physical activity and risk of large bowel cancer in the Framingham study. Cancer Res.

[40] Larsson SC, Rutegard J, Bergkvist L, Wolk A (2006). Physical activity, obesity, and risk of colon and rectal cancer in a cohort of Swedish men. Eur J Cancer.

[41] Lee IM, Paffenbarger RS (1994). Physical activity and its relation to cancer risk: a prospective study of college alumni. Med Sci Sports Exerc.

[42] Lee IM, Manson JE, Ajani U, Paffenbarger RS, Hennekens CH, Buring JE (1997). Physical activity and risk of colon cancer: the Physicians' health study (United States). Cancer Causes Control.

[43] Lee KJ, Inoue M, Otani T, Iwasaki M, Sasazuki S, Tsugane S (2007). Physical activity and risk of colorectal cancer in Japanese men and women: the Japan public health center-based prospective study. Cancer Causes Control.

[44] Thune I, Lund E (1996). Physical activity and risk of colorectal cancer in men and women. Br J Cancer.

[45] Morikawa T, Kuchiba A, Lochhead P, Nishihara R, Yamauchi M, Imamura Y (2013). Prospective analysis of body mass index, physical activity and colorectal cancer risk associated with β-catenin (CTNNB1) status. Cancer Res.

[46] Schmid D, Behrens G, Matthews CE, Leitzmann MF (2016). Physical activity and risk of colon cancer in diabetic and nondiabetic US adults. Mayo Clin Proc.

[47] Renehan AG, Tyson M, Egger M, Heller RF, Zwahlen M (2008). Body-mass index and incidence of cancer: a systematic review and meta-analysis of prospective observational studies. Lancet.

[48] Boyle T, Heyworth J, Bull F, McKerracher S, Platell C, Fritschi L (2011). Timing and intensity of recreational physical activity and the risk of subsite-specific colorectal cancer. Cancer Causes Control.

[49] Friedenreich CM, Cust AE (2008). Physical activity and breast cancer risk: impact of timing, type and dose of activity and population subgroup effects. Br J Sports Med.

[50] Quadrilatero J, Hoffman-Goetz L (2003). Physical activity and colon cancer. A systematic review of potential mechanisms. J Sports Med Phys Fitness.

[51] Bartram HP, Wynder EL (1989). Physical activity and colon cancer risk? Physiological considerations. Am J Gastroenterol.

[52] Roberts CK, Little JP, Thyfault JP (2013). Modification of insulin sensitivity and glycemic control by activity and exercise. Med Sci Sports Exerc.

[53] Kahn BB, Flier JS (2000). Obesity and insulin resistance. J Clin Investig.

[54] Burton AM, Hovick SR, Peterson SK (2012). Health behaviors in patients and families with hereditary colorectal cancer. Clin Colon Rectal Surg.

