# Supplementary material for: Effects of physical activity on colorectal cancer risk among family history and body mass index subgroups: a systematic review and meta-analysis
Source: BMC Cancer. 2018 Jan 11;18:71. doi: 10.1186/s12885-017-3970-5 (PMC5763991; doi:10.1186/s12885-017-3970-5)
Supplement: Additional file 1: Tables S1-S3. — PubMed search strategy for systematic review, Newcastle-Ottawa Scale for study quality assessment, and sensitivity analysis for individual study removal. (DOCX 21 kb) [file 12885_2017_3970_MOESM1_ESM.docx]

**Table S1.** PubMed search strategy for systematic review, completed July 11, 201

| **Concept** | **PubMed Search Terms** |
| --- | --- |
| Colorectal Cancer | (cancer OR neoplasm OR neoplasms OR carcinoma OR tumour OR tumor) AND (colon OR rectal OR intestine* OR rectum OR colorectal) |
| Physical Activity | (exercise OR "motor activity" OR "exercise therapy" OR "physical activity" OR walk OR bicycl* OR cycle OR cycling OR jog OR run) |
| Risk/Risk Factors | (“risk*” OR ‘’risk factor*’’) |
| High-Risk Subgroups | (polyp* OR polyps OR adenoma OR alcohol OR ethanol OR "folic acid" OR smoking OR tobacco OR obesity OR overweight OR BMI OR "body mass index" OR "family history" OR "genetic predisposition to disease" OR hereditary OR diet OR "diet therapy" OR "energy metabolism" OR "energy consumption" OR "energy intake") |

**Table S2:** Newcastle-Ottawa quality assessment scale for cohort and case-control studies

| **Study** | **Exposed cohort representative** | **Adequate unexposed selection** | **Exposure measure-ment** | **Outcome not present from start** | **Comparability of cohort^a^** | **Outcome assessment** | **Adequate period of follow-up^b^** | **Adequate follow-up of cohort^c^** | **Total** |
| --- | --- | --- | --- | --- | --- | --- | --- | --- | --- |
| **Cohort** |  |  |  |  |  |  |  |  |  |
| Ballard-Barbash, 1990 | 1 | 1 | 1 | 1 | 1 | 1 | 1 | 1 | 8 |
| Friedenreich, 2006 | 1 | 1 | 0.5 | 1 | 2 | 0.5 | 1 | 1 | 8 |
| Larsson, 2006 | 1 | 1 | 0 | 1 | 2 | 1 | 1 | 1 | 8 |
| Lee, I., 1994 | 0 | 1 | 0 | 1 | 2 | 1 | 1 | 1 | 7 |
| Lee, I., 1997 | 0 | 1 | 0 | 1 | 2 | 0 | 1 | 1 | 6 |
| Lee, K., 2007 | 1 | 1 | 0 | 1 | 2 | 1 | 1 | 1 | 8 |
| Morikawa, 2013 | 0 | 1 | 0 | 1 | 1 | 1 | 1 | 1 | 6 |
| Schmid, 2016 | 1 | 1 | 0 | 0 | 2 | 1 | 1 | 0 | 6 |
| Thune, 1996 | 1 | 1 | 0.5 | 0 | 2 | 1 | 1 | 1 | 7.5 |
|  |  |  |  |  |  |  |  |  |  |
| **Case-Control** | **Case-definition adequate** | **Cases representative** | **Control selection** | **Control definition** | **Comparability of cases and controls^a^** | **Exposure assessment** | **Same exposure assessment** | **Non-response rate^d^** | **Total** |
| Boutron-Ruault, 2009 | 1 | 0 | 1 | 0 | 2 | 0 | 1 | 1 | 6 |
| Huang, 2004 | 0 | 1 | 0 | 1 | 2 | 1 | 1 | 0 | 6 |
| Slattery, 1997 | 0 | 0 | 0 | 0 | 2 | 1 | 1 | 0 | 4 |
| Boyle, 2012 | 1 | 0 | 1 | 0 | 2 | 0 | 1 | 0 | 5 |
| Gerhardsson, 1990 | 1 | 1 | 1 | 0 | 2 | 0 | 1 | 1 | 7 |
| Hou, 2004 | 1 | 1 | 1 | 0 | 2 | 0 | 1 | 1 | 7 |
| Mao, 2003 | 0 | 1 | 1 | 1 | 1 | 0 | 1 | 1 | 6 |
| Parent, 2011 | 1 | 1 | 1 | 0 | 2 | 1 | 1 | 1 | 8 |
| Zhang, 2006 | 1 | 1 | 1 | 1 | 2 | 0 | 1 | 1 | 8 |

^a^ Comparability of cohort: 1 star if study controlledfor age, 2 stars for further controlling factors.

^b^ Adequate period of follow-up was defined as over 100 incident cases.

^c^ Adequate follow-up of cohort was defined as <20% lost to follow-up.

^d^ Adequate non-response rate was defined as <10% difference in response rate between cases and controls.

**Table S3:** Pooled overall relative risk estimates for physical activity and risk of colorectal cancer for high and low BMI subgroups with one study removed at a time

| **Study Removed** | **BMI group** | **OR (95% CI)** | ***I^2^* (%)** | **P_heterogeneity_** |
| --- | --- | --- | --- | --- |
| Gerhardsson, 1990 | Low | 0.75 (0.66-0.84) | 47.7 | 0.004 |
|  | High | 0.66 (0.54-0.82) | 88.9 | 0 |
| Ballard-Barbash, 1990 | Low | 0.74 (0.66-0.84) | 46.8 | 0.006 |
|  | High | 0.64 (0.51-0.79) | 89.6 | 0 |
| Lee I., 1994 | Low | 0.74 (0.66-0.83) | 47.9 | 0.003 |
|  | High | 0.65 (0.53-0.80) | 89.3 | 0 |
| Thune, 1996 | Low | 0.74 (0.66-0.83) | 44.8 | 0.009 |
|  | High | 0.63 (0.51-0.78) | 89.4 | 0 |
| Lee I., 1997 | Low | 0.73 (0.64-0.82) | 48.0 | 0.004 |
|  | High | 0.63 (0.51-0.78) | 89.1 | 0 |
| Slattery, 1997 | Low | 0.74 (0.65-0.84) | 51.1 | 0.002 |
|  | High | 0.66 (0.52-0.82) | 90.0 | 0 |
| Mao, 2003 | Low | 0.71 (0.63-0.81) | 39.4 | 0.024 |
|  | High | 0.64 (0.51-0.81) | 90.2 | 0 |
| Hou, 2004 | Low | 0.76 (0.67-0.87) | 52.3 | 0.003 |
|  | High | 0.76 (0.67-0.87) | 66.6 | 0 |
| Friedenreich, 2006 | Low | 0.74 (0.66-0.84) | 48.9 | 0.003 |
|  | High | 0.63 (0.51-0.78) | 89.5 | 0 |
| Larsson, 2006 | Low | 0.75 (0.67-0.84) | 45.3 | 0.007 |
|  | High | 0.65 (0.53-0.81) | 89.5 | 0 |
| Zhang, 2006 | Low | 0.76 (0.68-0.85) | 40.6 | 0.017 |
|  | High | 0.64 (0.51-0.80) | 89.6 | 0 |
| Lee K., 2007 | Low | 0.74 (0.66-0.84) | 47.0 | 0.005 |
|  | High | 0.62 (0.50-0.77) | 89.4 | 0 |
| Parent, 2011 | Low | 0.73 (0.64-0.83) | 51.8 | 0.002 |
|  | High | 0.64 (0.52-0.80) | 90.2 | 0 |
| Boyle, 2012 | Low | 0.74 (0.65-0.83) | 51.2 | 0.002 |
|  | High | 0.63 (0.51-0.78) | 89.4 | 0 |
| Morikawa, 2013 | Low | 0.73 (0.64-0.82) | 47.7 | 0.004 |
|  | High | 0.63 (0.52-0.76) | 86.3 | 0 |
| Schmid, 2016 | Low | 0.73 (0.64-0.83) | 49.9 | 0.002 |
|  | High | 0.63 (0.50-0.80) | 89.1 | 0 |
| **All Studies Included** | Low | 0.74 (0.66-0.83) | 47.9 | 0.003 |
|  | High | 0.65 (0.53-0.79) | 89.0 | 0 |
